# Skin Electrodes Based on TPU Fiber Scaffolds with Conductive Nanocomposites with Stretchability, Breathability, and Washability

**DOI:** 10.3390/mi15050598

**Published:** 2024-04-29

**Authors:** Zijia Zhao, Chaopeng Yang, Dongchan Li

**Affiliations:** School of Chemical Engineering and Technology, Hebei University of Technology, No. 5340, Xiping Road, Tianjin 300130, China; 202121503011@stu.hebut.edu.cn

**Keywords:** electronic skin, stretchable, breathable, electrospinning, intelligent wearable devices

## Abstract

In the context of an aging population and escalating work pressures, cardiovascular diseases pose increasing health risks. Electrocardiogram (ECG) monitoring presents a preventive tool, but conventional devices often compromise comfort. This study proposes an approach using Ag NW/TPU composites for flexible and breathable epidermal electronics. In this new structure, TPU fibers are used to support Ag NWs/TPU nanocomposites. The TPU fiber-reinforced Ag NW/TPU (TFRAT) nanocomposites exhibit excellent conductivity, stretchability, and electromechanical durability. The composite ensures high steam permeability, maintaining stable electrical performance after washing cycles. Employing this technology, a flexible ECG detection system is developed, augmented with a convolutional neural network (CNN) for automated signal analysis. The experimental results demonstrate the system’s reliability in capturing physiological signals. Additionally, a CNN model trained on ECG data achieves over 99% accuracy in diagnosing arrhythmias. This study presents TFRAT as a promising solution for wearable electronics, offering both comfort and functionality in long-term epidermal applications, with implications for healthcare and beyond.

## 1. Introduction

With the advent of the aging population and the simultaneous confrontation of individuals with elevated work pressures and a brisk pace of life, the incidence of cardiovascular diseases exhibits a discernible upward trend [1,2,3]. Cardiovascular diseases, constituting high-risk maladies, manifest not only through physical symptoms but also clinically present as anomalies in the ECG [4,5]. The long observation and accurate reflection of a human heart’s health condition through an electrocardiogram can play a preventive and diagnostic role, ensuring the wellbeing of individuals [6,7,8,9,10]. Traditional electrocardiogram monitoring devices are often cumbersome and require specific expertise to operate, making them less accommodating to users’ needs. However, flexible sensors utilizing electrodes with stretchability and breathability to gather human body information can cover specific skin areas for extended periods without causing discomfort. This allows for the continuous monitoring of vital signs, facilitating timely health assessments. Additionally, the integration of artificial intelligence (AI) has made the combination of flexible sensors and AI a prominent area of research.

To enhance signal quality, a compact interface should be established between the skin and the sensor [11,12,13]. Traditional electronic skin is manufactured on a substrate and then transferred onto the skin. Because the human body requires water evaporation to regulate temperature, the prolonged fixation of impermeable electronic devices on the skin may induce discomfort and even lead to adverse reactions [14,15,16,17]. Flexible porous materials, such as mesh-like membranes, elastomer microfoams, and resilient textiles, have been widely studied as breathable platforms for stretchable electronic devices. The interconnected micro-pores in these materials provide exceptional breathability, greatly enhancing comfort during wear [18,19,20,21,22]. Various nanomaterials, such as silver nanoparticles, Ag NWs, and thin metal coatings, have been incorporated into porous substrates to fabricate conductive electrodes that are stretchable [23,24,25,26,27]. However, manufacturing flexible devices on porous substrates still requires specialized equipment and complex procedures [28,29,30]. Another design employs elastic membranes to construct breathable epidermal electronic devices [31,32,33,34]. Elastic materials are not ideal barriers for gas molecules due to their abundant free volume [35,36,37,38,39]. Additionally, if elastic materials contain a large number of hydrophilic groups, they can actively participate in the transport of water molecules, significantly enhancing the breathability of the elastic membrane. Therefore, elastomers have been widely utilized in manufacturing stretchable thin-film devices with open mesh designs that possess breathable characteristics [24]. An approach involves embedding metal nanowires into elastomers to manufacture breathable electrodes with deformability [40,41,42,43]. Another advantage of the design is its low mechanical stiffness, facilitating a close connection with highly textured skin [44,45,46]. However, these enviable characteristics unfortunately come at the expense of reduced mechanical toughness [47,48,49,50]. Due to insufficient stiffness for direct manipulation, carefully designed procedures are required to transfer prepared devices onto the human body. Additionally, removing such devices from the skin often entails applying excessive force, posing a high risk of structural damage [51,52,53]. Therefore, ultrathin breathable devices are typically considered as disposable electronic tattoos for short-term implementation.

Traditional research on arrhythmia classification aims to automate the methods used by human experts in order to identify and classify arrhythmias from ECG signals. The main challenges in traditional arrhythmia classification research lie in the diversity of arrhythmia types, as well as the presence of overlap and confusion, necessitating the optimization and improvement of classification algorithms and feature extraction to enhance accuracy and stability [54,55]. With the continuous development of computers, researchers have explored the direction of machine learning technologies. Due to the rapid advancement of AI technologies, machine learning and related techniques have been widely applied to arrhythmia recognition and classification, achieving notable results. Traditional arrhythmia classification methods have limitations in extracting deep features from ECG signal data. By employing deep learning techniques such as neural networks, researchers aim to achieve more accurate and automated arrhythmia diagnosis [56,57]. CNN and recurrent neural network (RNN) are widely used learning models in arrhythmia classification research. Researchers also employ various preprocessing and data augmentation techniques to enhance model performance and generalization ability. Deep learning techniques hold great potential for arrhythmia classification and are expected to become important tools for future automatic arrhythmia diagnosis [58,59,60]. A CNN, as one of the deep learning models, is specifically designed to handle data with grid-like structures, such as images, videos, and audio. Compared to traditional neural networks, CNNs excel in capturing local features and spatial relationships when processing such data. Moreover, CNNs are applied in the morphological analysis of physiological signals due to their unique positional and shift-invariant capturing capabilities. ECG signals contain local features related to arrhythmias, such as QRS complexes, ST segments, and T waves, which are independent of the overall shape and size of the signal [51]. Therefore, CNNs are suitable for arrhythmia classification tasks and have achieved promising results in this field.

Ag NWs embedded in an elastic film form a flexible nano-composite electrode with inherent gas permeability. The electrospun TPU solution produces a flexible and porous substrate, allowing for convenient operation and repeated use. Laser cutting is employed for patterning the nano-composite electrode. This electrode exhibits low resistance (180.1 mΩ/sq) and moderate stretchability. The overall architecture of the electrode in the electrospun fiber ensures high steam permeability during use, providing a comfortable experience. Stable electrical performance is maintained even after multiple washing cycles, meeting long-term hygiene requirements. Finally, a flexible system for ECG detection is developed based on TFRAT. A CNN is built and trained. This neural network can automatically analyze the ECG signals obtained from the flexible system. In conclusion, comparing TFRAT with previous electrodes, it has certain advantages (Table S1).

## 2. Materials and Methods

The following chemicals were used: ethylene glycol from Tianjin benchmark Chemical Reagent Co., Ltd. (Tianjin, China); polyvinylpyrrolidone (PVP) from Wuxi Yatai United Chemical Co., Ltd. (Tianjin, China); silver nitrate (AgNO_3_) and anhydrous ethanol from Tianjin fengchuan Chemical Reagent Research Co., Ltd. (Tianjin, China); TPU (soft35A 12P) from BASF, Ludwigshafen, Germany; and N,N-Dimethylformamide (DMF) from Shanghai Mayer Biochemical Technology Co., Ltd. (Shanghai, China).

Ag NWs were synthesized via the polyol reduction method in our laboratory. During the synthesis of the Ag NW precursor solution, 0.63 g of AgNO_3_ and 1 g of PVP were added to a 100 mL ethylene glycol solution that was stirred until they were completely dissolved. Subsequently, 225 µL of 88 mM CuCl_2_ solution was added. The mixture was then immediately transferred to a preheated reactor and kept at 130 °C for 12 h, resulting in Ag NWs that were approximately 86 µm in length and around 70 nm in diameter (as shown in Figure S1).

The Ag NWs were added to DMF and stirred until they were uniformly dispersed. Then, TPU particles were added to the mixture and stirred until the TPU particles were completely dissolved. The prepared and fresh Ag NW/TPU was used. The prepared Ag NW/TPU solution was poured into a polytetrafluoroethylene (PTFE) container, and it dried and formed a film at room temperature.

Then, 20 g of DMF and 20 g of THF were mixed separately, after which 10 g of TPU pellets was poured into the mixed solution, followed by stirring for 4 h at 60 °C. The TPU solution was then drawn into a 20 mL plastic syringe and secured on an injection pump with a flow rate of 5 mL/h. The distance between the needle tip and the collector is set at 16 cm, with a potential difference of 16 kV between the needle tip and the collector.

### Characterization

The electronic universal testing machine (CMT 6104 100 N sensor, Shenzhen new think measurement technology Co., Ltd., Shenzhen, China) was employed to assess the mechanical properties of fiber fabrics and the tensile properties of TFRAT. Scanning electron microscopy (SEM 7610F, Tokyo, Japan) images were used to observe the micromorphology of Ag NWs and TFRAT. The four-probe resistance tester (M-3) was utilized to measure the sheet resistance of the electrode. The stream permeability test involves sealing a glass jar filled with deionized water using the sample and placing it in an environmental chamber. The weight of the glass jar was periodically measured using an analytical balance with an accuracy of ±0.1 mg. To assess long-term storage stability, the samples were placed in an environmental chamber for 15 consecutive days, during which resistance was recorded. Washing tests were performed by immersing the samples in tap water. The samples were placed into a beaker filled with distilled water, and the beaker was then placed on a stirring hotplate and stirred for 1 h to simulate the washing conditions of a washing machine. After the washing cycle, the samples were spread out and left to air-dry naturally indoors. Subsequently, the resistance of the samples was measured using a four-probe resistance meter, and the changes before and after washing were recorded. The resistance changes after multiple wash cycles can be used to evaluate the washability of the flexible electrodes. In addition to using distilled water, detergent can also be added for comparison purposes. The skin electrode’s contact impedance was measured using a VICTOR LCR meter. The original ECG and EMG signals were amplified via the Intan RHD2000 amplifier and then sampled using the Intan RHD USB interface board.

## 3. Results and Discussion

**Figure 1:**
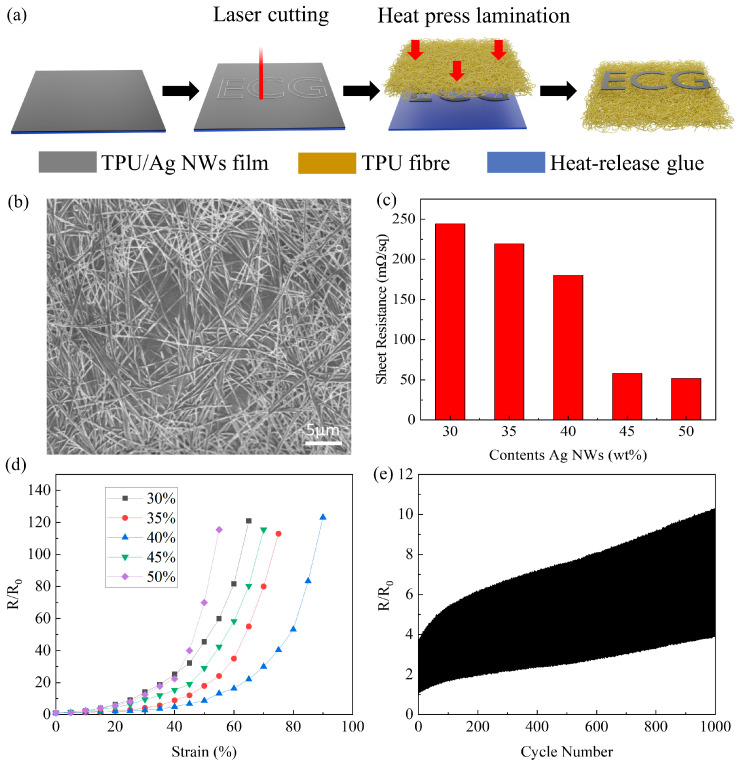
(**a**) The fabrication process of TFRAT. (**b**) SEM images of TFRAT with 40% Ag NWs content. (**c**) Electrical properties of the Ag NW/TPU film with varying Ag NW content. (**d**) Normalized resistance vs. tensile resistance for conductive nanocomposites with different Ag NW contents. (**e**) Change in the resistance of the TFRAT with 40 wt % Ag NWs during 1000 stretch–relaxation cycles relative to 30% strain.

Figure 1a schematically illustrates the entire manufacturing process of TFRAT. Firstly, the previously prepared AgNW/TPU composite films were transferred onto a thermal release adhesive. After natural drying, the film’s thickness was approximately 10 µm. Next, the Ag NW/TPU film was cut via laser cutting according to the pattern designed in CAD drawings, removing excess parts afterward. Finally, the Ag NW/TPU composite film was attached to a TPU fiber membrane substrate using a hot press. The composite film was heated to 120 °C, and, after 10 s, the adhesive on the heat-release glue did not exhibit activity, allowing the removal of the heat-release tape. The polymer scaffold prepared through electrospinning exhibits excellent stretchability. As observed in the stress–strain curve, the elongation at break of the polymer scaffold is 412%, and Young’s modulus is 1 MPa (as shown in Figure S2). It possesses high conductivity, breathability, and washability, making it highly suitable for applications in electronic textiles.

Concerning electrical performance, the SEM image in Figure 1b reveals that Ag NWs are uniformly embedded into the TPU substrate and distributed on the surface of the TPU. These Ag NWs form a conductive network within the Ag NW/TPU composite film. Figure 1c indicates that, with an increase in Ag NW content in the composite film, the sheet resistance of the Ag NW/TPU composite film gradually decreases, demonstrating that the overlap of the Ag NWs’ conductive network increases with the increase in Ag NW content. Specifically, the sheet resistance is 244.3 mΩ/sq at 30 wt %, 219.3 mΩ/sq at 35 wt %, 180.1 mΩ/sq at 40 wt %, 57.8 mΩ/sq at 45 wt %, and 51.7 mΩ/sq at 50 wt %. From Figure S3 and Figure 1b, it can be observed that the internal conductive network of Ag NWs is denser, increasing from 40% to 45%, which is also the reason for the decrease in resistance. The sharp decrease in sheet resistance may be due to crossing a threshold as a result of the addition of Ag NWs, resulting in a significant increase in the contact interface between Ag NWs.

The relationship between the resistance of TFRAT and tensile strain is illustrated in Figure 1d. Specifically, the normalized resistance is 2.8 at 30% strain and increases to 123.1 at 90% strain. TFRAT improves its stretchability by completely embedding Ag NWs in the TPU film. The initially reduced sensitivity at increased loading is likely due to the diminished influence of individual junctions within the densely interconnected network. Conversely, an excess of Ag NWs tends to result in pronounced stiffening effects on the corresponding nanocomposites [61]. In terms of durability, Figure 1e illustrates the evolution of resistance after 1000 stretching cycles at 30% strain. As observed in the stress–strain curve (Figure S4), the elongation at break of TFRAT is 453%.

**Figure 2:**
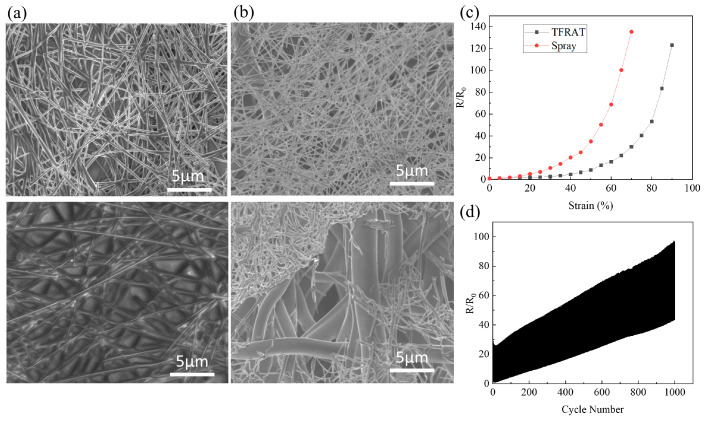
(**a**) SEM images of TFRAT before (**top**) and after (**bottom**) tensile strain at 30%; (**b**) SEM images of TPU fibers before (**top**) and after (**bottom**) tensile strain at 30%; (**c**) the relationship between normalized resistance and tensile strain for TFRAT and spray-coated electrodes; (**d**) change in the resistance of the nanocomposite with sprayed Ag NWs during 1000 stretch–relaxation cycles to 30% strain.

Correspondingly, the resistance of electrodes formed by directly spraying Ag NWs onto TPU fibers undergoes rapid changes with variations in tensile strain. Figure 2a illustrates that the irreversible changes in resistance are primarily attributed to the inconsistent deformation between Ag NWs and TPU, leading to the sliding of nanowires and damage to the connected network [35,62]. In Figure 2c, the resistance of the spray-coated electrodes increases sharply by 10.6 times at 30% strain and further escalates to 135.3 times at 70%, substantially below the tensile performance of TFRAT. The degradation in electrical performance is primarily associated with the fracture of suspended Ag NWs at micro-pores, whereas TFRAT enhances its tensile capability by embedding Ag NWs completely within the TPU film. Regarding durability, Figure 2d illustrates the evolution of resistance after 1000 cycles of stretching at 30% strain. For the sprayed electrodes, the nominal resistance increases by a factor of 43.5 in the relaxed state, whereas for TFRAT, the nominal resistance increases by a factor of 4.1 (as shown in Figure 1e). TFRAT exhibits good mechanical durability in practical applications.

**Figure 3:**
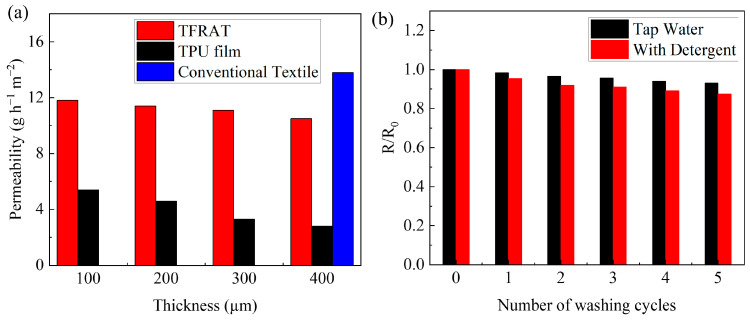
(**a**) Steam permeability for the TFRAT, TPU film, and conventional textile; (**b**) the nominal resistance of TFRAT varies with washing cycles.

Simultaneously, breathable design is crucial for the comfortable wear of skin-adherent electronic products. In Figure 3a shows the water vapor permeability for several wearable materials. Conventional TPU thin films have a low permeability of 2.8 g h^−1^ m^−2^ at 400 μm, which blocks the transepidermal water loss of the body (~5–10 g h^−1^ m^−2^) [63]. The porous structure of the TPU fiber membrane facilitates the permeation of water vapor, with TFRAT achieving a breathability of 11.2 g h^−1^ m^−2^ at 400 μm, exhibiting slightly lower breathability than traditional textiles (13.8 g h^−1^ m^−2^). Accordingly, TFRAT represents an attractive electronic material for long-term epidermal applications. Furthermore, in practical applications, TFRATs typically feature specific patterns; in other words, the TPU/Ag NWs composite material does not completely cover the flexible and breathable substrate. The areas that are not covered by the TPU/Ag NW composite material may further enhance its breathability. Therefore, this experiment also discusses the relationship between the coverage ratio of the conductive film on the substrate and its corresponding breathability. The results, as shown in Figure S5, indicate that when the coverage ratio is 25%, breathability is 17.7 g h^−1^ m^−2^, 16.8 g h^−1^ m^−2^ at 50%, 16.0 g h^−1^ m^−2^ at 75%, and 11.4 g h^−1^ m^−2^ at 100%. Even when the coverage ratio of the transfer-printed electrode reaches 100%, its overall breathability can still meet the requirements of human skin.

In practical applications, conductive microfabrics need to exhibit stable electrical performance. TFRAT demonstrates high stability when stored for an extended period under environmental conditions, maintaining high conductivity (as shown in Figure S6). In terms of wash resistance, the conductive fabric was initially evaluated through simulated washing tests. Surprisingly, TFRAT shows a decrease in resistance during the washing process (as shown in Figure 3b), and this is possibly due to the partial removal of PVP surfactants during washing, resulting in a reduction in contact resistance between Ag NWs [64]. The relatively stable resistance indicates its good wash resistance, enabling its application in a wide range of fields, surpassing disposable devices. In addition, in this experiment, a washing test was also conducted on the spray-coated electrodes for comparison purposes, as shown in Figure S7. It can be observed that the washing performance of the spray-coated electrodes is very poor. The resistance of the spray-coated electrodes is 3.12 times the initial resistance after the first wash, 12 times after repeated washing, and 268 times after the third wash. This may be because the electrode fabrication process results in different distributions of conductive materials, thereby affecting the washing performance of the electrodes. As shown in Figure S8, the microstructure of the transfer-printed electrodes does not show significant changes after washing; the Ag NWs remain well embedded within the TPU. This structure ensures that it is less susceptible to damage inflicted on the conductive network due to external influences. In contrast, significant changes in morphology are observed for the spray-coated electrodes after washing. The conductive network formed by Ag NWs on the surface of the spray-coated electrodes is severely disrupted after washing. Although some Ag NWs still adhere to the fibers, providing some conductivity, this is insufficient for meeting the requirements of the daily use of electrodes.

**Figure 4:**
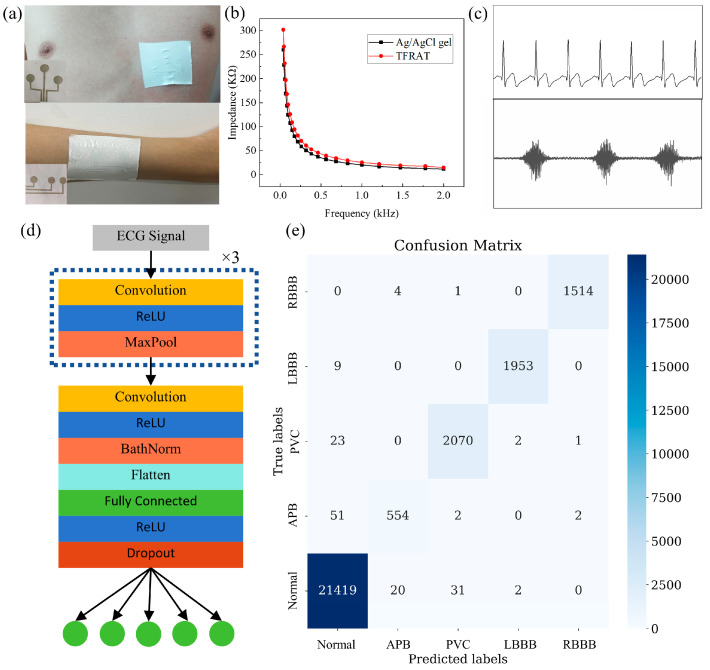
(**a**) ECG recording (**top**) and EMG recording (**bottom**); inset: as-prepared epidermal patches consisting of sensing electrodes and interconnects; (**b**) skin–electrode contact impedance as a function of frequency for TFRAT and commercial Ag/AgCl gel electrode; (**c**) ECG (**top**) and EMG (**bottom**) measurements of a person measured using TFRAT; (**d**) the CNN intelligent algorithm model for four typical arrhythmia classification and diagnostics; (**e**) confusion matrix of the intelligent algorithm model for the four types of arrhythmia diagnosis.

Therefore, corresponding epidermal sensing patches were prepared (Figure S9), and the manufactured sensing patches were adhered to the skin using breathable surgical tape (Figure 4a). According to the impedance analysis shown in Figure 4b, the skin contact impedance of TFRAT is slightly higher overall than that of gel electrodes, especially in the low-frequency region where the difference between the two is more pronounced. However, as the frequency increases, this difference begins to diminish. This is possibly because gel electrodes possess adhesive properties, allowing them to better adhere to the skin and reduce contact impedance. In contrast, TFRAT requires external forces to adhere better to the skin. However, this can be improved by adjusting the applied pressure and increasing skin moisture, among other methods. As shown in Figure 4c, high-quality ECG signals and EMG signals were obtained from the TFRAT electrode. The unique features in these ECG waveforms allow for quantitative heart rate calculations and useful diagnostics for cardiac diseases. To further illustrate the quality of the ECG signals collected via TFRAT, we instructed the test subjects to attach TFRAT to their left chest and then to go for a run. The collected ECG signals during the running process are shown in Figure S10. Due to the stretching of the skin, increased heart rate, and the influence of sweat during the running process, significant changes occurred in the ECG signals. However, TFRAT was still able to collect high-quality ECG signals. Additionally, we conducted tests on TFRAT by applying it to the human body and keeping it attached for 24 h, even during showering, without detachment. After removal, there were no noticeable changes on the skin surface, indicating that TFRAT exhibits excellent biocompatibility and does not cause skin irritation or rashes (Figure S11). Overall, TFRAT is well suited as an epidermal electrode, reliably capturing physiological electrical signals.

Recently, CNNs integrated with flexible devices have been utilized for the analysis of human gestures, demonstrating significant potential in healthcare and intelligent systems. ECG signals can assist cardiac experts in analyzing various diseases such as atrial fibrillation and early ventricular contractions. In the real-time and long-term monitoring of patients, it is impractical for cardiac experts to offer continuous real-time assessments of ECG signals. Therefore, there is a need to develop a computer-assisted arrhythmia analysis system. In addition to neural networks, large datasets of ECG recordings are necessary to achieve accurate results. The widely used ECG dataset is the MIT-BIH Arrhythmia Database. In this work, we developed a prediction model based on the MIT-BIH Arrhythmia Database, consisting of a 9-layer CNN. This model can detect four different arrhythmia types, including left bundle branch block (LBBB), right bundle branch block (RBBB), atrial premature beats (APBs), and premature ventricular contractions (PVCs).

When CNNs are used for image recognition, as the number of network layers increases, complex high-level features gradually decompose into basic low-level features, and the combination of numerous low-level features serves as the basis for distinguishing between different categories of images. The convolutional layer (convolution) is used to extract features from input data by performing convolution operations with convolutional kernel sliding over the input data to generate output feature maps. The max pooling layer (max pooling) reduces the spatial size of feature maps while preserving the most significant features by selecting the maximum value within each pool. The rectified linear unit (ReLU) and other activation functions are introduced into CNN to enhance their non-linear capabilities, aiding in the further improvement of network performance. The batch normalization layer (batch norm) accelerates the training process of neural networks and helps mitigate issues such as vanishing or exploding gradients by normalizing the activations of each layer. The flatten layer (flatten) converts multidimensional input data into a one-dimensional vector, and it is typically used before connecting to fully connected layers. The fully connected layer (full connection) flattens the multi-channel features generated by different convolutional kernels to obtain the final classification result. The dropout layer (dropout) is employed during training to randomly drop neurons, reducing overfitting.

The structure of the prediction model is illustrated in Figure 4d, with the first three layers composed of a CNN layer, an ReLU layer, and a max pooling layer. The final convolutional layer is followed by a batch normalization layer and a flattening layer to transform the output of the convolutional layers from a 2D matrix to a 1D matrix. Next, there is a fully connected layer with 128 nodes, followed by a dropout layer (dropout rate of 0.2) to prevent overfitting. Finally, the output passes through a fully connected layer with five nodes for the classification output. Overall, the model’s structure is designed to extract features from inputs through convolution and pooling operations, and it then classifies them through fully connected layers.

To fully utilize the label of each heartbeat, 99 signal points are taken forward and 200 signal points are taken backward from the R peak, forming a complete heartbeat. In the preprocessing stage, 70% of the entire dataset is used for training, and the remaining samples are used as test samples. As shown in the confusion matrix in Figure 4e, the prediction accuracy on the test dataset exceeds 99%, indicating the potential of this system to assist doctors in diagnosing arrhythmias.

## 4. Conclusions

In this study, we have introduced the fabrication of TFRAT, a type of skin electronic characterized by both stretchability and breathability. In this architecture, the TPU fiber membrane serves as the support for the material composed of Ag NW/TPU. The shear lamination method enables the scalable production of Ag NW/TPU film nanocomposite electrodes, which exhibit excellent conductivity, stretchability, and mechanical durability. The structure of TFRAT demonstrates high vapor permeability, providing a breathability advantage in skin-related sensing applications. Its robust electrical properties ensure long-term usability, even after repeated washing cycles. Leveraging TFRAT, a flexible system has been developed for detecting electrocardiogram signals and analyzing ECG data using CNN. This research shows promising potential for future wearable devices and healthcare applications.

While our study showcases promising advancements in wearable electronics, it is essential to acknowledge some limitations and identify future research opportunities. One limitation lies in the sensitivity of TFRAT to environmental factors such as humidity and temperature variations, which may affect its performance in real-world scenarios. Additionally, the fabrication process of TFRAT could be further optimized to enhance scalability and reduce production costs. Future studies should explore methods to mitigate the sensitivity of TFRAT to environmental factors, possibly through the integration of protective coatings or alternative materials. Furthermore, improvements in fabrication techniques could lead to the development of TFRAT variants with enhanced properties, such as increased stretchability or improved breathability. Moreover, investigating the integration of TFRAT into multifunctional wearable systems for health monitoring beyond ECG, such as sweat analysis or motion tracking, presents exciting avenues for research. In conclusion, while TFRAT holds significant promise for wearable electronics and healthcare applications, addressing its limitations and pursuing future research directions will be crucial for realizing its full potential.

## Data Availability

The data presented in this study are available on request from the corresponding author.

## Supplementary Materials

The following supporting information can be downloaded at: https://www.mdpi.com/article/10.3390/mi15050598/s1. Figure S1: (a) SEM image of as-synthesized Ag NWs by using polyol reduction method; Figure S2: Stress-strain curve of conductive microtextile under uniaxial tensile stretching; Figure S3: SEM images of TFRAT with 45% Ag NWs content; Figure S4: Stress-strain curve of TFRAT under uniaxial tensile stretching; Figure S5: Steam permeability for TFRAT of different coverage; Figure S6: Long-term storage stability at the ambient temperature with different relative humidity levels. The resistance is fairly stable under both dry and humid air conditions; Figure S7: The water washing performance diagram of TFRAT and spray electrode; Figure S8: Surface SEM plot of the sample after wash cycles (a) TFRAT; (b) spray electrode; Figure S9: TFRAT diagrammatic sketch; Figure S10: ECG signals of the running state; Figure S11: After wearing TFRAT for 24 h (a) before removal; (b) after removal. Table S1: A summary of stretchable and breathable conductors.

## References

[1] Bayoumy K., Gaber M., Elshafeey A., Mhaimeed O., Dineen E.H., Marvel F.A., Martin S.S., Muse E.D., Turakhia M.P., Tarakji K.G. (2021). Smart wearable devices in cardiovascular care: Where we are and how to move forward. Nat. Rev. Cardiol..

[2] Hannun A.Y., Rajpurkar P., Haghpanahi M., Tison G.H., Bourn C., Turakhia M.P., Ng A.Y. (2019). Cardiologist-level arrhythmia detection and classification in ambulatory electrocardiograms using a deep neural network. Nat. Med..

[3] Murray C.J.L., Lopez A.D. (1997). Alternative projections of mortality and disability by cause 1990–2020: Global Burden of Disease Study. Lancet.

[4] Chen G., Xiao X., Zhao X., Tat T., Bick M., Chen J. (2021). Electronic Textiles for Wearable Point-of-Care Systems. Chem. Rev..

[5] Zhang Y., Zhang T., Huang Z., Yang J. (2022). A New Class of Electronic Devices Based on Flexible Porous Substrates. Adv. Sci..

[6] Li C.H., Mu J.H., Song Y.J., Chen S., Xu F. (2023). Highly aligned cellulose/polypyrrole composite nanofibers via electrospinning and in situ polymerization for anisotropic flexible strain sensor. ACS Appl. Mater. Interfaces.

[7] Huang H., Wu N., Liu H., Dong Y., Han L., Wan S., Dou G., Sun L. (2021). Directional Sweat Transport and Breathable Sandwiched Electrodes for Electrocardiogram Monitoring System. Adv. Mater. Interfaces.

[8] Li J., Pan X., Zhang Y., Liu Y., Wang C., Wan Y., Tao J., Bao R., Pan C. (2023). Ultrathin breathable and stretchable electronics based on patterned nanofiber composite network. Mater. Today Nano.

[9] Mohseni Taromsari S., Shi H.H., Salari M., Eskandarian L., Habibpour S., Yu A., Park C.B., Naguib H.E. (2023). Electromechanical properties and physiological sensing enhancement of an in-situ assembled 3-D nanostructure: A comprehensive effect assessment of tannic acid treated 2-D Ti3C2Tx and 1-D graphene nanoribbon (GnR). Carbon.

[10] Liang X.P., Zhu M.J., Li H.F., Dou J.X., Jian M.Q., Xia K.L., Li S., Zhang Y.Y. (2022). Hydrophilic, breathable, and washable graphene decorated textile assisted by silk sericin for integrated multimodal smart wearables. Adv. Funct. Mater..

[11] Zhou Y.L., Cao S.T., Wang J., Zhu H.Y., Wang J.C., Yang S.N., Wang X., Kong D.S. (2018). Bright Stretchable Electroluminescent Devices based on Silver Nanowire Electrodes and High-k Thermoplastic Elastomers. ACS Appl. Mater. Interfaces.

[12] Wang Y.H., Yin L., Bai Y.Z., Liu S.Y., Wang L., Zhou Y., Hou C., Yang Z.Y., Wu H., Ma J.J. (2020). Electrically compensated, tattoo-like electrodes for epidermal electrophysiology at scale. Sci. Adv..

[13] Huang Y.A., Wu H., Zhu C., Xiong W.N., Chen F.R., Xiao L., Liu J.P., Wang K.X., Li H.Y., Ye D. (2021). Programmable robotized ‘transfer-and-jet’ printing for large, 3D curved electronics on complex surfaces. Int. J. Extrem. Manuf..

[14] Wang P., Sun G., Yu W., Li G., Meng C., Guo S. (2022). Wearable, ultrathin and breathable tactile sensors with an integrated all-nanofiber network structure for highly sensitive and reliable motion monitoring. Nano Energy.

[15] Wang P., Yu W., Li G., Meng C., Guo S. (2023). Printable, flexible, breathable and sweatproof bifunctional sensors based on an all-nanofiber platform for fully decoupled pressure–temperature sensing application. Chem. Eng. J..

[16] Wen Y., Zhao R., Yin X., Shi Y., Fan H., Zhou Y., Tan L. (2020). Antibacterial and Antioxidant Composite Fiber Prepared from Polyurethane and Polyacrylonitrile Containing Tea Polyphenols. Fibers Polym..

[17] Zhang J.-H., Li Z., Xu J., Li J., Yan K., Cheng W., Xin M., Zhu T., Du J., Chen S. (2022). Versatile self-assembled electrospun micropyramid arrays for high-performance on-skin devices with minimal sensory interference. Nat. Commun..

[18] Ji S., Wan C., Wang T., Li Q., Chen G., Wang J., Liu Z., Yang H., Liu X., Chen X. (2020). Water-Resistant Conformal Hybrid Electrodes for Aquatic Endurable Electrocardiographic Monitoring. Adv. Mater..

[19] Sempionatto J.R., Lin M., Yin L., De la paz E., Pei K., Sonsa-ard T., de Loyola Silva A.N., Khorshed A.A., Zhang F., Tostado N. (2021). An epidermal patch for the simultaneous monitoring of haemodynamic and metabolic biomarkers. Nat. Biomed. Eng..

[20] Sun T., Hui J., Zhou L., Lin B., Sun H., Bai Y., Zhao J., Mao H. (2022). A low-cost and simple-fabricated epidermal sweat patch based on “cut-and-paste” manufacture. Sens. Actuators B Chem..

[21] Wu H., Yang G., Zhu K., Liu S., Guo W., Jiang Z., Li Z. (2020). Materials, Devices, and Systems of On-Skin Electrodes for Electrophysiological Monitoring and Human–Machine Interfaces. Adv. Sci..

[22] Yang S., Chen Y.C., Nicolini L., Pasupathy P., Sacks J., Su B., Yang R., Sanchez D., Chang Y.F., Wang P. (2015). “Cut-and-Paste” Manufacture of Multiparametric Epidermal Sensor Systems. Adv. Mater..

[23] Chang K., Guo M., Pu L., Dong J., Li L., Ma P., Huang Y., Liu T. (2023). Wearable nanofibrous tactile sensors with fast response and wireless communication. Chem. Eng. J..

[24] Ding C., Wang J., Yuan W., Zhou X., Lin Y., Zhu G., Li J., Zhong T., Su W., Cui Z. (2022). Durability Study of Thermal Transfer Printed Textile Electrodes for Wearable Electronic Applications. ACS Appl. Mater. Interfaces.

[25] Zhou Y.L., Wang S.L., Yin J.Y., Wang J.J., Manshaii F., Xiao X., Zhang T.Q., Bao H., Jiang S., Chen J. (2024). Flexible Metasurfaces for Multifunctional Interfaces. ACS Nano.

[26] Zhou Y.L., Yin L.T., Xiang S.F., Yu S., Johnson H.M., Wang S.L., Yin J.Y., Zhao J., Luo Y., Chu P.K. (2024). Unleashing the Potential of MXene-Based Flexible Materials for High-Performance Energy Storage Devices. Adv. Sci..

[27] Zhu C., Xu Z.Y., Hou C., Lv X.D., Jiang S., Ye D., Huang Y.A. (2024). Flexible, monolithic piezoelectric sensors for large-area structural impact monitoring via MUSIC-assisted machine learning. Struct. Health Monit..

[28] Jost V. (2018). Packaging related properties of commercially available biopolymers—An overview of the status quo. Express Polym. Lett..

[29] Kang K.S., Jee C., Bae J.-H., Jung H.J., Kim B.J., Huh P. (2018). Effect of soft/hard segments in poly (tetramethylene glycol)-Polyurethane for water barrier film. Prog. Org. Coat..

[30] Wang Q.L., Zhang G.N., Zhang H.Y., Duan Y.Q., Yin Z.P., Huang H.A. (2021). High-Resolution, Flexible, and Full-Color Perovskite Image Photodetector via Electrohydrodynamic Printing of Ionic-Liquid-Based Ink. Adv. Funct. Mater..

[31] Yin R., Yang S., Li Q., Zhang S., Liu H., Han J., Liu C., Shen C. (2020). Flexible conductive Ag nanowire/cellulose nanofibril hybrid nanopaper for strain and temperature sensing applications. Sci. Bull..

[32] Zhang L., Jiang F., Wang L., Feng Y., Yu D., Yang T., Wu M., Petru M. (2022). High Performance Flexible Strain Sensors Based On Silver Nanowires/thermoplastic Polyurethane Composites for Wearable Devices. Appl. Compos. Mater..

[33] Zhu H.-W., Gao H.-L., Zhao H.-Y., Ge J., Hu B.-C., Huang J., Yu S.-H. (2020). Printable elastic silver nanowire-based conductor for washable electronic textiles. Nano Res..

[34] Zou X., Xue J., Li X., Chan C.P.Y., Li Z., Li P., Yang Z., Lai K.W.C. (2023). High-Fidelity sEMG Signals Recorded by an on-Skin Electrode Based on AgNWs for Hand Gesture Classification Using Machine Learning. ACS Appl. Mater. Interfaces.

[35] Ma T., Lin Y., Ma X.H., Zhang J.X., Li D.C., Kong D.S. (2023). Stretchable, breathable, and washable epidermal electrodes based on microfoam reinforced ultrathin conductive nanocomposites. Nano Res..

[36] Wang Y.H., Tang T.Y., Xu Y., Bai Y.Z., Yin L., Li G., Zhang H.M., Liu H.C., Huang Y.A. (2021). All-weather, natural silent speech recognition via machine-learning-assisted tattoo-like electronics. npj Flex. Electron..

[37] Xiong W.N., Zhu C., Guo D.L., Hou C., Yang Z.X., Xu Z.Y., Qiu L., Yang H., Li K., Huang Y.A. (2021). Bio-inspired, intelligent flexible sensing skin for multifunctional flying perception. Nano Energy.

[38] Zhuang M.Q., Yin L., Wang Y.H., Bai Y.Z., Zhan J., Hou C., Yin L.T., Xu Z.Y., Tan X.H., Huang Y.A. (2021). Highly Robust and Wearable Facial Expression Recognition via Deep-Learning-Assisted, Soft Epidermal Electronics. Research.

[39] Huang Y.A., Zhu C., Xiong W.N., Wang Y., Jiang Y.G., Qiu L., Guo D.L., Hou C., Jiang S., Yang Z.X. (2022). Flexible smart sensing skin for "Fly-by-Feel" morphing aircraft. Sci. China-Technol. Sci..

[40] Xiong W.N., Feng H., Liwang H.S., Li D., Yao W.B., Duolikun D., Zhou Y.L., Huang Y.A. (2022). Multifunctional Tactile Feedbacks Towards Compliant Robot Manipulations via 3D-Shaped Electronic Skin. IEEE Sens. J..

[41] Yin Z.P., Huang Y.A., Yang H., Chen J.K., Duan Y.Q., Chen W. (2022). Flexible electronics manufacturing technology and equipment. Sci. China-Technol. Sci..

[42] Zhou Y.L., Qu Y.P., Yin L.T., Cheng W.N., Huang Y.A., Fan R.H. (2022). Coassembly of elastomeric microfibers and silver nanowires for fabricating ultra-stretchable microtextiles with weakly and tunable negative permittivity. Compos. Sci. Technol..

[43] Bai Y.Z., Yin L.T., Hou C., Zhou Y.L., Zhang F., Xu Z.Y., Li K., Huang Y.A. (2023). Response Regulation for Epidermal Fabric Strain Sensors via Mechanical Strategy. Adv. Funct. Mater..

[44] Zhi C.W., Shi S., Zhang S., Si Y.F., Yang J.Q., Meng S., Fei B., Hu J.L. (2023). Bioinspired all-fibrous directional moisture-wicking electronic skins for biomechanical energy harvesting and all-range health sensing. Nano-Micro Lett..

[45] Kim Y.S., Mahmood M., Lee Y., Kim N.K., Kwon S., Herbert R., Kim D., Cho H.C., Yeo W.H. (2019). All-in-One, Wireless, Stretchable Hybrid Electronics for Smart, Connected, and Ambulatory Physiological Monitoring. Adv. Sci..

[46] Qiao Y., Li X., Jian J., Wu Q., Wei Y., Shuai H., Hirtz T., Zhi Y., Deng G., Wang Y. (2020). Substrate-Free Multilayer Graphene Electronic Skin for Intelligent Diagnosis. ACS Appl. Mater. Interfaces.

[47] Cui T., Qiao Y., Li D., Huang X., Yang L., Yan A., Chen Z., Xu J., Tan X., Jian J. (2023). Multifunctional, breathable MXene-PU mesh electronic skin for wearable intelligent 12-lead ECG monitoring system. Chem. Eng. J..

[48] Chen F.R., Gai M.X., Sun N.N., Xu Z.Y., Liu L., Yu H.Y., Bian J., Huang Y.A. (2023). Laser-driven hierarchical “gas-needles” for programmable and high-precision proximity transfer printing of microchips. Sci. Adv..

[49] Zhou Y.L., Cheng W.N., Bai Y.Z., Hou C., Li K., Huang Y.A. (2023). Rise of flexible high-temperature electronics. Rare Met..

[50] Qu Y.P., Zhou Y.L., Luo Y., Liu Y., Ding J.F., Chen Y.L., Gong X., Yang J.L., Peng Q., Qi X.S. (2024). Universal paradigm of ternary metacomposites with tunable epsilon-negative and epsilon-near-zero response for perfect electromagnetic shielding. Rare Met..

[51] Chen E., Jiang J., Su R., Gao M., Zhu S., Zhou J., Huo Y. (2020). A new smart wristband equipped with an artificial intelligence algorithm to detect atrial fibrillation. Heart Rhythm.

[52] Wang F., Chen J.W., Cui X.H., Liu X.N., Chang X.H., Zhu Y.T. (2022). Wearable Ionogel-Based Fibers for Strain Sensors with Ultrawide Linear Response and Temperature Sensors Insensitive to Strain. ACS Appl. Mater. Interfaces.

[53] Li J.-W., Lee J.C.-M., Chuang K.-C., Chiu C.-W. (2023). Photocured, highly flexible, and stretchable 3D-printed graphene/polymer nanocomposites for electrocardiography and electromyography smart clothing. Prog. Org. Coat..

[54] Himori S., Sakata T. (2022). Free-standing conductive hydrogel electrode for potentiometric glucose sensing. RSC Adv..

[55] Horev Y.D., Maity A., Zheng Y., Milyutin Y., Khatib M., Yuan M., Suckeveriene R.Y., Tang N., Wu W., Haick H. (2021). Stretchable and highly permeable nanofibrous sensors for detecting complex human body motion. Adv. Mater..

[56] Gopi C., Vinodh R., Sambasivam S., Obaidat I.M., Kim H.J. (2020). Recent progress of advanced energy storage materials for flexible and wearable supercapacitor: From design and development to applications. J. Energy Storage.

[57] Han J.K., Yang J.K., Gao W.W., Bai H. (2021). Ice-templated, large-area silver nanowire pattern for flexible transparent electrode. Adv. Funct. Mater..

[58] Ma Y.J., Zhang Y.C., Cai S.S., Han Z.Y., Liu X., Wang F.L., Cao Y., Wang Z.H., Li H.F., Chen Y.H. (2020). Flexible hybrid electronics for digital healthcare. Adv. Mater..

[59] Qin Z., Chen X., Yin Y., Ma G., Jia Y., Deng J., Pan K. (2019). Flexible janus electrospun nanofiber films for wearable triboelectric nanogenerator. Adv. Mater. Technol..

[60] Qiao Y., Gou G., Shuai H., Han F., Liu H., Tang H., Li X., Jian J., Wei Y., Li Y. (2022). Electromyogram-strain synergetic intelligent artificial throat. Chem. Eng. J..

[61] Lin Y., Li Q.S., Ding C., Wang J.Y., Yuan W., Liu Z.Y., Su W.M., Cui Z. (2022). High-resolution and large-size stretchable electrodes based on patterned silver nanowires composites. Nano Res..

[62] Tian B., Fang Y., Liang J., Zheng K., Guo P., Zhang X., Wu Y., Liu Q., Huang Z., Cao C. (2022). Fully Printed Stretchable and Multifunctional E-Textiles for Aesthetic Wearable Electronic Systems. Small.

[63] Pinnagoda J., Tupkek R.A., Agner T., Serup J. (1990). Guidelines for transepidermal water loss (TEWL) measurement. Contact Dermat..

[64] Zhao H., Zhou Y., Cao S., Wang Y., Zhang J., Feng S., Wang J., Li D., Kong D. (2021). Ultrastretchable and Washable Conductive Microtextiles by Coassembly of Silver Nanowires and Elastomeric Microfibers for Epidermal Human–Machine Interfaces. ACS Mater. Lett..

