# Supplementary material for: Skin Electrodes Based on TPU Fiber Scaffolds with Conductive Nanocomposites with Stretchability, Breathability, and Washability
Source: Micromachines (Basel). 2024 Apr 29;15(5):598. doi: 10.3390/mi15050598 (PMC11122800; doi:10.3390/mi15050598)
Supplement: Supplementary file 1 [file micromachines-15-00598-s001.zip › Supporting information .pdf]

Supporting information

Skin Electrodes Based on TPU Fiber Scaffolds with Conductive Nanocomposites with Stretchability, Breathability, and Washability

Zijia Zhao, Chaopeng Yang * and Dongchan Li *

School of Chemical Engineering and Technology, Hebei University of Technology, No. 5340, Xiping Road, Tianjin 300130, China; 202121503011@stu.hebut.edu.cn

* Correspondence: 036300106@163.com (C.Y.); dongchanli@hebut.edu.cn (D.L.)

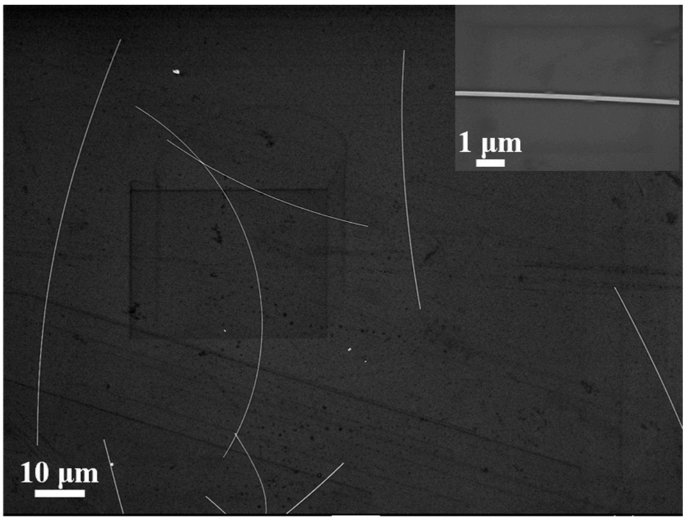

Figure S1 SEM image of as-synthesized Ag NWs by using polyol reduction method.

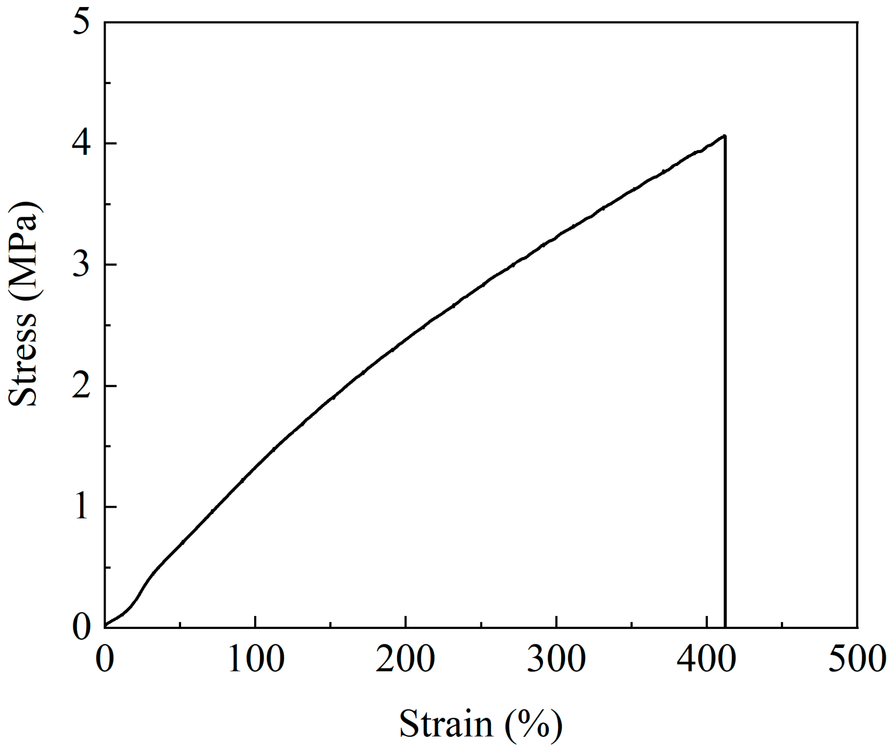

Figure S2 Stress-strain curve of TPU fibers under uniaxial tensile stretching

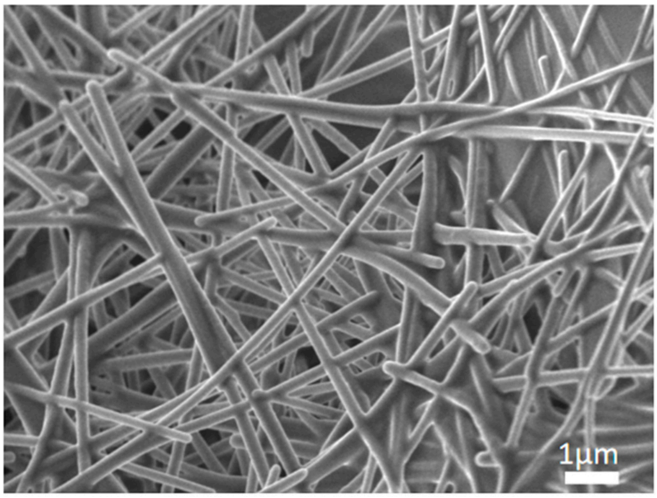

Figure S3 SEM images of TFRAT with 45% Ag NWs content

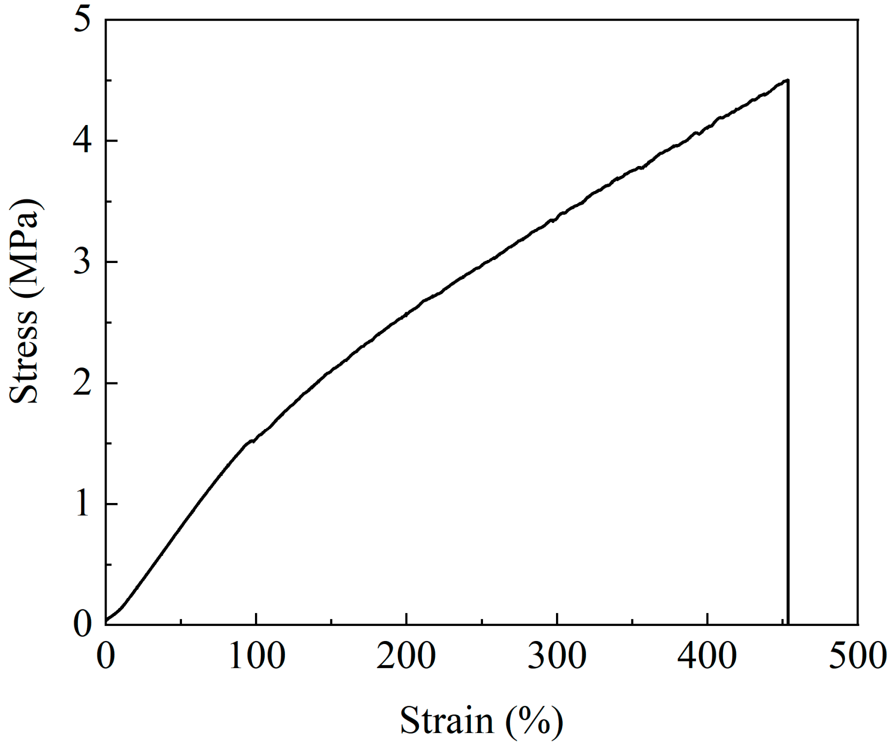

Figure S4 Stress-strain curve of TFRAT under uniaxial tensile stretching

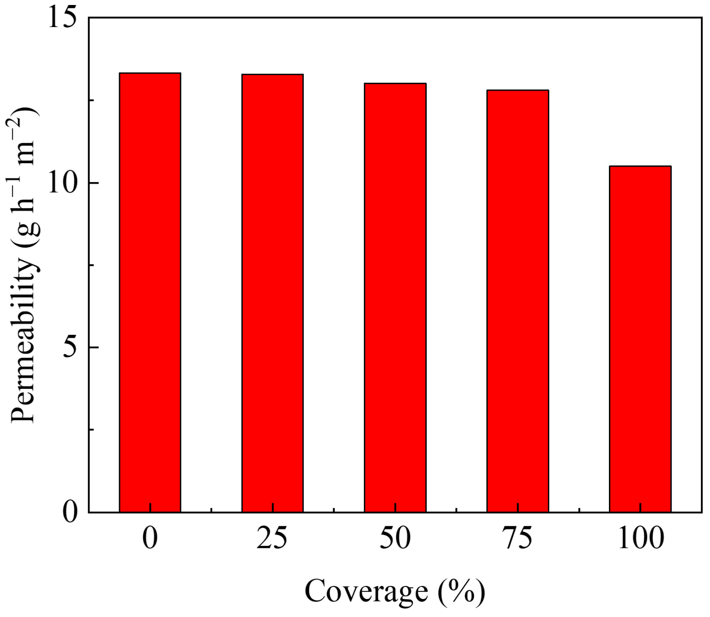

Figure S5 Steam permeability for TFRAT of different coverage

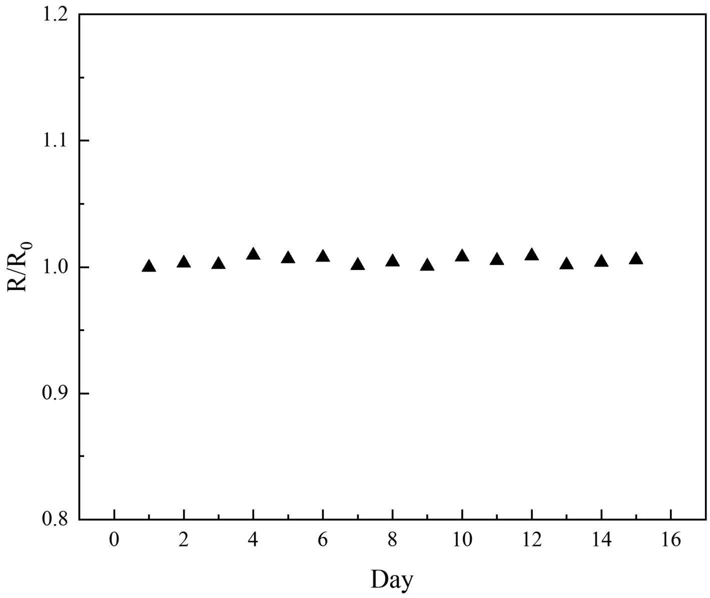

Figure S6 Long-term storage stability at the ambient temperature with different relative humidity levels. The resistance is fairly stable under both dry and humid air conditions

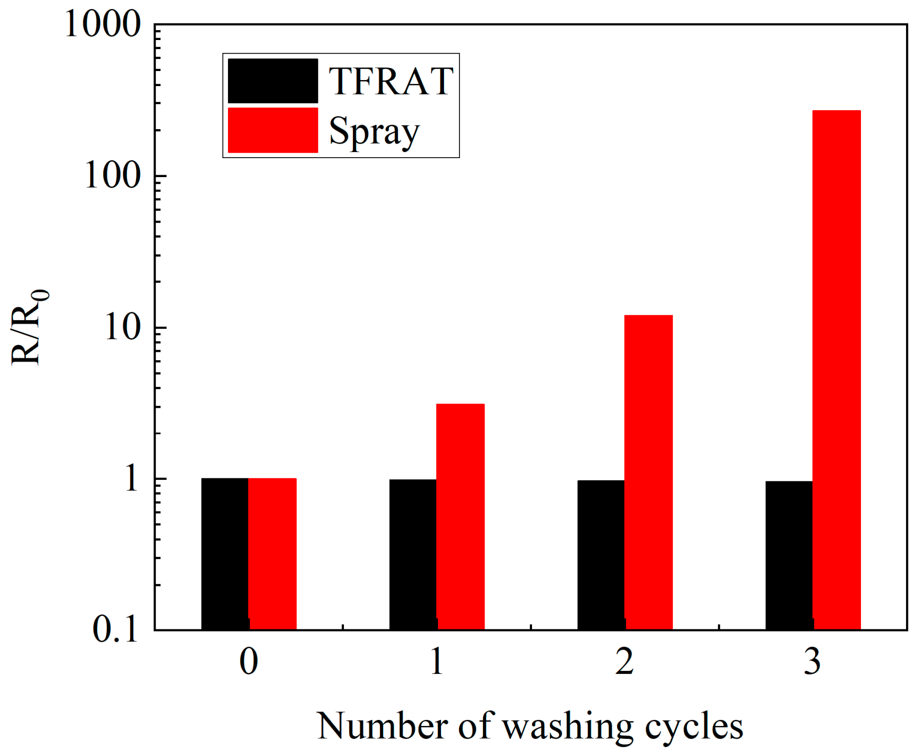

Figure S7 The water washing performance diagram of TFRAT and spray electrode

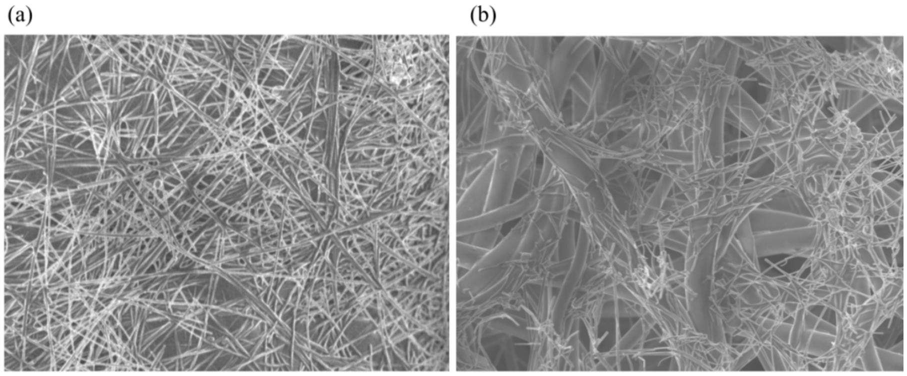

Figure S8 Surface SEM plot of the sample after wash cycles(a) TFRAT; (b) spray electrode

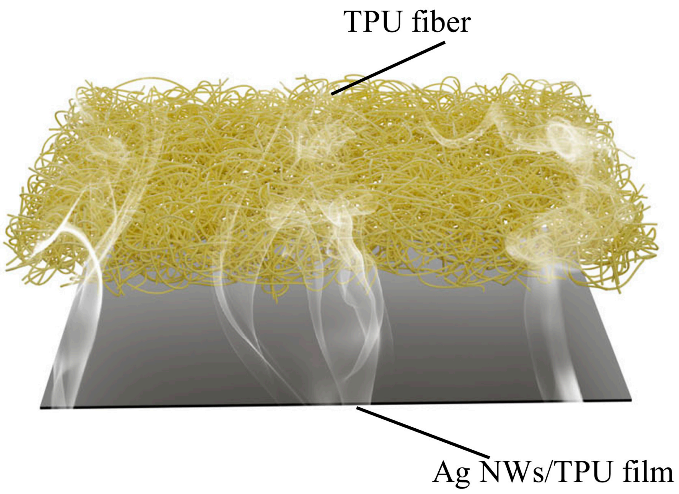

Figure S9 TFRAT diagrammatic sketch

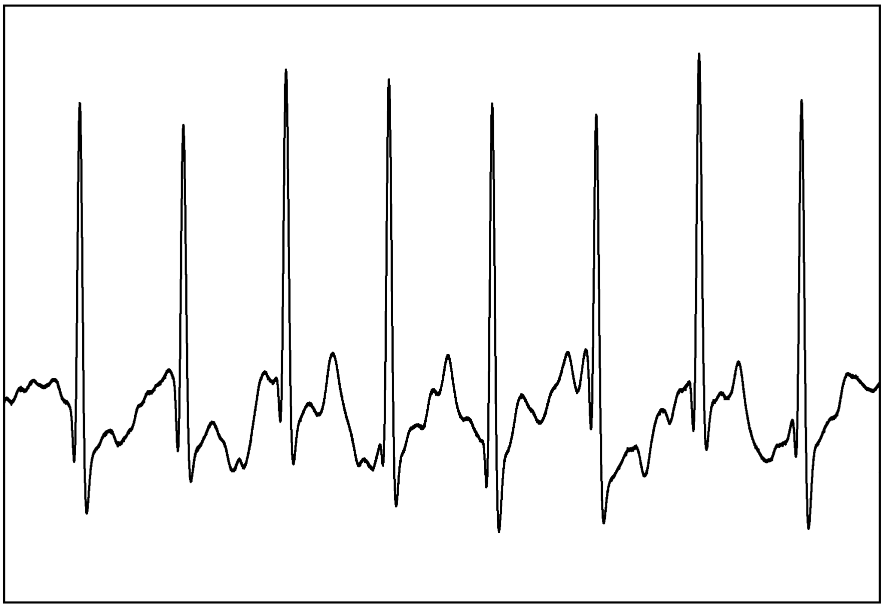

Figure S10 ECG signals of the running state

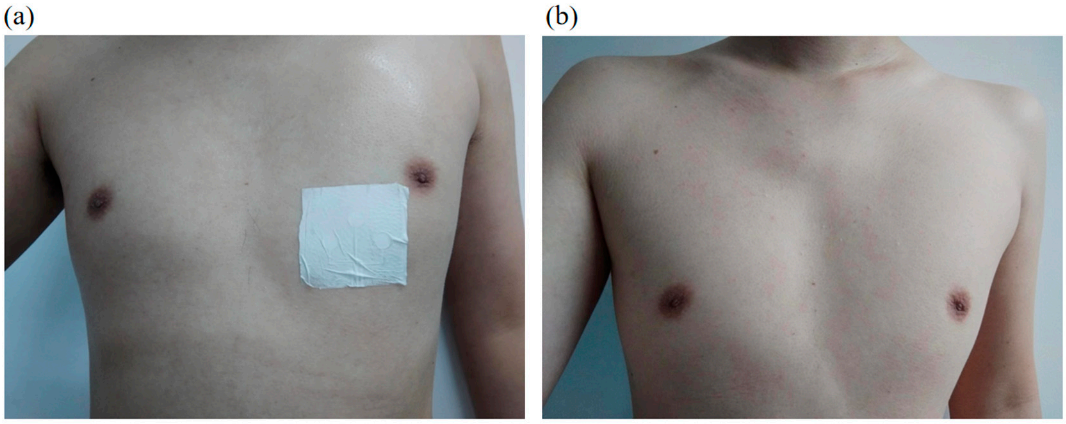

Figure S11 After wearing TFRAT for 24h (a) before removal; (b) after removal

Table S1 A summary of stretchable and breathable conductors.

Stretchable Conductor	Square resistance	Stretchability	Washability	Algorithm	Refs
TPAT	180.1 mΩ/sq	90%	Yes	CNN	This Work
Ag NW-TPE membrane	11.87 Ω/sq	62%	No	None	[1]
Au film/fluorine rubber fiber	22.8 Ω/sq	170%	NO	None	[2]
Au and Au/PU Nanomesh	6.09 Ω/sq	50%	No	CNN	[3]
AgNWs/PDMS film	0.481 Ω/sq	54%	Yes	LDA	[4]
Porous Ag NW/TPU	7.4 Ω/sq	15%	No	No	[5]
Ag NW/TPU nanofiber	4 Ω/sq	50%	Yes	XGboost	[6]
microfoam reinforced ultrathin conductive nanocomposite	0.45 Ω/sq	80%	Yes	None	[7]

References

1. Yang, X.Q., Li, L.H., Wang, S.Q., Lu, Q.F., Bai, Y.Y., Sun, F.Q., Li, T., Li, Y., Wang, Z.H., Zhao, Y.Y., Shi, Y.X., Zhang, T.: Ultrathin, Stretchable, and Breathable Epidermal Electronics Based on a Facile Bubble Blowing Method. Advanced Electronic Materials. **6**, 11 (2020).
2. Li, Q.S., Ding, C., Yuan, W., Xie, R.J., Zhou, X.M., Zhao, Y., Yu, M., Yang, Z.J., Sun, J., Tian, Q., Han, F., Li, H.F., Deng, X.P., Li, G.L., Liu, Z.Y.: Highly Stretchable and Permeable Conductors Based on Shrinkable Electrospun Fiber Mats. Advanced Fiber Materials. **3**, 302-311 (2021).
3. Qiao, Y., Gou, G., Shuai, H., Han, F., Liu, H., Tang, H., Li, X., Jian, J., Wei, Y., Li, Y., Xie, C., He, X., Liu, Z., Song, R., Zhou, B., Tian, H., Yang, Y., Ren, T.-L., Zhou, J.: Electromyogram-strain synergetic intelligent artificial throat. Chemical Engineering Journal. **449**, (2022).
4. Zou, X., Xue, J., Li, X., Chan, C.P.Y., Li, Z., Li, P., Yang, Z., Lai, K.W.C.: High-Fidelity sEMG Signals Recorded by an on-Skin Electrode Based on AgNWs for Hand Gesture Classification Using Machine Learning. ACS Applied Materials & Interfaces. **15**, 19374-19383 (2023).
5. Zhou, W.X., Yao, S.S., Wang, H.Y., Du, Q.C., Ma, Y.W., Zhu, Y.: Gas-Permeable, Ultrathin, Stretchable Epidermal Electronics with Porous Electrodes. Acs Nano. **14**, 5798-5805 (2020).
6. Wang, Y.F., Wang, J., Cao, S.T., Kong, D.S.: A stretchable and breathable form of epidermal device based on elastomeric nanofibre textiles and silver nanowires. Journal of Materials Chemistry C. **7**, 9748-9755 (2019).
7. Ma, T., Lin, Y., Ma, X.H., Zhang, J.X., Li, D.C., Kong, D.S.: Stretchable, breathable, and washable epidermal electrodes based on microfoam reinforced ultrathin conductive nanocomposites. Nano Research. **16**, 10412-10419 (2023).
